# Breast Cancer Anti-Estrogen Resistance 4 (BCAR4) Drives Proliferation of IPH-926 lobular Carcinoma Cells

**DOI:** 10.1371/journal.pone.0136845

**Published:** 2015-08-28

**Authors:** Ton van Agthoven, Lambert C. J. Dorssers, Ulrich Lehmann, Hans Kreipe, Leendert H. J. Looijenga, Matthias Christgen

**Affiliations:** 1 Department of Pathology, Erasmus MC Cancer Institute, Rotterdam, the Netherlands; 2 Institute of Pathology, Hannover Medical School, Hannover, Germany; Taipei Medical University, TAIWAN

## Abstract

**Background:**

Most breast cancers depend on estrogenic growth stimulation. Functional genetic screenings in *in vitro* cell models have identified genes, which override growth suppression induced by anti-estrogenic drugs like tamoxifen. Using that approach, we have previously identified Breast Cancer Anti-Estrogen Resistance 4 (*BCAR4)* as a mediator of cell proliferation and tamoxifen-resistance. Here, we show high level of expression and function of *BCAR4* in human breast cancer.

**Methods:**

*BCAR4* mRNA expression was evaluated by (q)RT-PCR in a panel of human normal tissues, primary breast cancers and cell lines. A new antibody raised against C78-I97 of the putative BCAR4 protein and used for western blot and immunoprecipitation assays. Furthermore, siRNA-mediated gene silencing was implemented to study the function of BCAR4 and its downstream targets ERBB2/3.

**Results:**

Except for placenta, all human normal tissues tested were *BCAR4*-negative. In primary breast cancers, *BCAR4* expression was comparatively rare (10%), but associated with enhanced proliferation. Relative high *BCAR4* mRNA expression was identified in IPH-926, a cell line derived from an endocrine-resistant lobular breast cancer. Moderate *BCAR4* expression was evident in MDA-MB-134 and MDA-MB-453 breast cancer cells. BCAR4 protein was detected in breast cancer cells with ectopic (ZR-75-1-*BCAR4*) and endogenous (IPH-926, MDA-MB-453) *BCAR4* mRNA expression. Knockdown of *BCAR4* inhibited cell proliferation. A similar effect was observed upon knockdown of ERBB2/3 and exposure to lapatinib, implying that *BCAR4* acts in an ERBB2/3-dependent manner.

**Conclusion:**

*BCAR4* encodes a functional protein, which drives proliferation of endocrine-resistant breast cancer cells. Lapatinib, a clinically approved EGFR/ERBB2 inhibitor, counteracts *BCAR4*-driven tumor cell growth, a clinical relevant observation.

## Introduction

Estrogen receptor (ER)-positive mammary carcinomas account for the vast majority of breast cancer (BC) cases. They depend on estrogenic growth stimulation. Anti-hormone therapy is the corner stone in the clinical management of ER-positive BC [1]. Adjuvant therapy with anti-estrogenic drugs, such as tamoxifen, presumably suppressing proliferation of remnant BC cells, prolongs survival and reduces mortality [2]. However, after prolonged anti-hormone therapy, BC cells can escape from growth suppression. This becomes apparent by disease progression or tumor recurrences that do not respond to endocrine therapy any longer. Occasionally, acquired endocrine resistance is accompanied by conversion of the ER status, illustrating that estrogenic growth stimulation has become dispensable [3].

The molecular mechanisms underlying endocrine resistance are diverse [4, 5]. Many candidate genes responsible for endocrine resistance have been reported. Several of these genes are clinically relevant [6]. Their mRNA or protein levels correlate with tamoxifen-resistance and/or tumor aggressiveness [7]. We have previously isolated Breast Cancer Anti-estrogen Resistance 4 (*BCAR4*), a gene that can override tamoxifen-induced growth suppression *in vitro* [8–11]. In brief, *BCAR4* was identified by a functional genetic screening in the ER-positive and estrogen-dependent BC cell line ZR-75-1 [8]. These cells were infected with retroviruses containing >1.10^7^ independent cDNAs, representing expression libraries from human brain, placenta, HeLa cervical carcinoma cells or mouse embryo cells. Subsequently, ZR-75-1 cells were selected for their ability to proliferate in the presence of tamoxifen. When insertion and expression of a cDNA allowed for the formation of a proliferating cell colony, the inserted gene was identified by PCR and nucleotide sequence analysis. *BCAR4*, which was solely recovered from the placenta-derived cDNA library, was the most frequently identified gene in tamoxifen-resistant ZR-75–1 colonies [8]. Retroviral transduction of ZR-75-1 with only a *BCAR4* expression construct yielded the same phenotype [10]. ZR-75-1-*BCAR4* cells proliferated despite hormone deprivation or exposure to various anti-estrogens [10]. Functional characterization of this genetically engineered cell model revealed that *BCAR4* acts independently from ER-associated signal transduction and enhances cell proliferation *via* activation of the ERRB2/3 pathway, even if ERBB2 is expressed at low levels [9].

Apart for these findings from *in vitro* models, the physiological function of *BCAR4* is ill defined. The *BCAR4* gene is well conserved in primates [10]. Distant *BCAR4* orthologues also exist in other placental species, but not in the rodents mouse and rat [10]. Meta-analyses of Gene Expression Omnibus (GEO) data sets have indicated a tissue-specific expression of *BCAR4* in the placenta in all stages of development [10]. Moreover, *BCAR4* is expressed in matured and fertilized bovine oocytes [12]. *BCAR4* is annotated as a long non-coding RNA (LncRNA) [13, 14]. However, we have observed that a frameshift mutation, disrupting the predicted open reading frame, abrogates the pro-proliferative activity of *BCAR4* [8]. This implies the existence of a putative BCAR4 protein.

In clinical BC specimens, *BCAR4* mRNA is detectable in approximately 10–27% of cases, depending on the tumor collection and the assay [9]. In patients treated with tamoxifen for advanced disease, higher *BCAR4* mRNA levels are associated with an aggressive tumor phenotype and reduced progression-free survival [9]. Human BC cell lines with high endogenous *BCAR4* expression have not been described, so far [10]. This has limited studies into *BCAR4* function to genetically engineered cells, whose physiological relevance is uncertain.

Here, we report on the high endogenous *BCAR4* mRNA and protein expression in IPH-926, a cell line derived from an endocrine-resistant lobular BC [15–18]. Our results show that *BCAR4* encodes for a functional protein, which is critical for cell proliferation. Lapatinib, a clinically approved ERBB2/EGFR inhibitor [19], counteracts *BCAR4*-driven tumor cell growth.

## Materials and Methods

### Cell lines and culture conditions

Human BC cell lines were cultured as described previously [11, 10, 9]. Cell lines were obtained from ATCC (Manassas, U.S.A.). Estrogen-dependent ZR-75-1 BC cells [20] were a kind gift of R.J.B. King, ICRF, London. Transfected or retroviral transduced ZR-75-1 cells with ectopic overexpression of *BCAR1*, *BCAR3*, *BCAR4* and *EGFR* have been described previously [11, 10]. For EGFP-positive cells, ZR-75-1 was transfected with expression vector pEGFP-N1 (Clontech, Westburg B.V., Leusden, the Netherlands) or an EGFP-N1-*BCAR4* fusion construct. All cell lines were authenticated by short tandem repeat (STR) profiling with the Powerplex6 system (Promega,Leiden, the Netherlands). The IPH-926 cell line was additionally authenticated by PCR-based detection of its unique *CDH1* 241ins4 frameshift mutation, as described previously [15].

### Primary BC specimens and human normal tissues

Formalin-fixed paraffin-embedded (FFPE) primary, pre-treatment BC specimens and human normal tissues were retrieved from the tissue archive of the Hannover Medical School according to the guidelines of the local ethics committee (Ethics Committee of the Hannover Medical School, Department of Forensic Medicine). Written informed consent of tissue donors was waived for archival pathological specimens by the Ethics Committee of the Hannover Medical School, Department of Forensic Medicine. All specimens were made anonymous for scientific purposes. Clinicopathological characteristics are reported in Table 1. For expression analyses, tumor tissue was macro-dissected by means of sampling two 1.4 mm (diameter) cores. Extraction of total RNA and cDNA synthesis were performed as described previously [21].

**Table 1:** Characteristics of primary, pre-treatment BCs.

	cases	percent
**all samples**	96	100
**age**		
<60	24	25
≥60	72	75
**histological type**		
ductal	53	55
lobular	43	45
**histological grade**		
G1/2	67	70
G3	29	30
**pT stage**		
T1	49	51
T2	32	33
T3/4	15	16
**pN stage**		
pN0	51	53
pN1+	33	34
pNx	12	13
**estrogen receptor**		
negative	14	15
positive	82	85
**progesterone receptor**		
negative	39	41
positive	57	59
**ERBB2**		
0, 1+	88	92
2+/FISH-negative	3	3
3+	5	5
**EGFR**		
negative	94	98
positive	2	2
**CK5/14**		
negative	86	90
positive	10	10
**Ki67 LI**		
0–14	47	49
15–34	33	34
35–100	16	17

### Generation of the C78- I97 polyclonal anti-BCAR4 antibody

A polyclonal anti-BCAR4 antibody, termed C78-I97, was prepared by immunization of two rabbits with a combination of two human BCAR4-specific synthetic peptides (H2N-CTVDENLQKTTRLR-CONH2 and H2N-CIRKSGSLQGTTEPSM-CONH2) corresponding to BCAR4 amino acids 78–93 and 97–110 (Eurogentec, Seraing, Belgium). Immunization and production of the antibodies was commercially performed at Eurogentec, which is authorised to manufacture biologics at its site in Liège, Belgium under the Authorisation No.: 1285, issued by The Belgian Ministry of Health. The institutional animal care and use committee: Ethic Committee from CER group. Final bleed of the animals was performed with an overdose of barbiturates and exsanguination to reduce suffering in compliance with known and recognized scientific protocols for many years. Animal facilities are in compliance with standard of housing (2013/63/EU). In addition—Compliance with standards of staff training (manipulations, observations, administrations) (CA / 2013/05/29). -Compliance with internal standards for the quality of animal feed (much stricter than legislation) - Compliance with ISO9001 standards for tracking and data archiving.

For purification of anti-BCAR4 antibodies, His-tagged-BCAR4 protein was produced in BL21 bacteria, solubilized in urea buffer using sonification, and purified on NiNTA beads. The eluate was concentrated on Amicon Ultra centrifugal filters. For antibody purification, approximately 4x10^10^ magnetic beads (Sphero, CMS-30-10, 2.5% weight/volume, 3.28 μm) were washed, re-suspended in NaPI pH 6.2 buffer and activated with Sulfo-NHS and EDC (Pierce, Rockford, IL, U.S.A.) for 20 min at room temperature. After washing with MES pH 5.0 buffer, the beads were mixed with 100 μl of BCAR4 protein diluted in 5 ml of MES (2-(N-morpholino) ethanesulfonic acid) buffer and mixed for 2 hours at room temperature. Beads were recovered by centrifugation, washed with PBS-TBN (PBS, 0.1% BSA, 0.02% Tween-20, 0.05% sodium azide) to block the coupling and to remove unbound BCAR4 protein. Beads with bound BCAR4 were stored in PBS-TBN at 4°C. Crude antibody serum (100 μl) was purified with Melon Gel IgG purification kit following the protocol of the manufacturer (Pierce/Thermo Scientific, Rockford IL, U.S.A.). 100 μl of the approximately 10-fold diluted flow-through was mixed with BCAR4-loaded beads and incubated for 1–2 hours. Unbound Ig was washed away with PBS-T and bound Ig was eluted with 0.1 M glycine-HCl pH 2.6 buffer. Eluates were immediately neutralized by adding 1 M Tris-HCl pH 9.0. Fractions containing specific antibodies directed against BCAR4 protein were pooled and supplemented with BSA and 1 volume of glycerol and were stored at -20°C.

### Western blot and immunoprecipitation

Immunoprecipitation and western blotting were performed as described previously [9, 15]. Membranes were probed with antibodies against BCAR4 (C78-I97, see above), EGFR (clone 2.1E1, Zytomed Systems, Berlin, Germany), ERBB2 (clone 4B5, Ventana, Tucson, AZ, U.S.A.), and *β*-actin (clone AC15, Acris, Hiddenhausen, Germany) and Anti-Flag M1 (Sigma, Zwijndrecht, the Netherlands).

### Quantitative real-time RT-PCR ((q)RT-PCR)

Extraction of total RNA and cDNA synthesis were performed as described previously [21, 15]. Quantitative assessment of gene expression normalized to the housekeeping gene *GUSB* was performed with Platinum Taq DNA polymerase (Invitrogen, Karlsruhe, Germany), Sybr Green I (Invitrogen) and the QuantiTect^®^ *BCAR4* primer assay (Qiagen, Hilden, Germany) on an ABI Prism 7700 system (Applied Biosystems, Foster City, U.S.A.). For monitoring of siRNA-mediated inhibition, *HPRT*, *B2M* and *PBGD* were employed as reference genes for normalization.

### Immunohistochemistry

For immunohistochemical characterization of primary BCs, FFPE tissue sections were mounted on poly-l-lysine coated slides and were deparaffinized and rehydrated conventionally. Antigenic retrieval was achieved by pressure cooking at 125°C in 10 mM citric acid (pH 6) for 3 min. Endogenous peroxidase was blocked with 0.3% (v/v) H_2_O_2_. Slides were incubated with primary antibodies and the ZytoChem-Plus HRP kit (Zytomed, Berlin, Germany) was used for detection of the immune reaction. Antibody clone designations, dilutions and scoring systems have been described elsewhere [22]. In accordance with the definition of Cheang and colleagues, the luminal-B molecular subtype was defined as ER-α (designated below as ER)-positive or PR-positive, CK5/14-negative, EGFR-negative, ERBB2-negative, Ki67 LI ≥15 [23, 24]. Expression of ERBB2 was defined according to the clinical assay (0, 1+, 2+, 3+), as recommended in the HercepTest (DAKO, Hamburg Germany) [25].

### Cell proliferation assay

Cells were seeded into 96-well plates at a density of 5000 cells/well. The next day, a mixture containing siRNA dilution and the transfection reagent DharmaFect3 (Dharmacon, Thermo Scientific, Etten-Leur, the Netherlands) was added to the wells. Final concentrations were 25 nM for the siRNA and 0.1% for the transfection reagent. In experiments using siRNAs against *EGFR* media were supplemented with 10 ng/ml EGF (Roche Diagnostics, Almere, the Netherlands). For each condition, at least six replicates were included. All siRNAs were On TARGETplus-SMARTpools, each consisting of three different oligonucleotides directed against *EGFR* (L-003114-00-0005), *ERBB2* (L-003126-00-005), *ERBB3* (L-003127-00-0005) or *ERBB4* (L003128-00-0005) (Dharmacon). The siRNAs directed against *BCAR4* (siBCAR4, AM 16708A, IDs 276004, 268474 and 268475) were purchased from Life Technologies (Darmstadt, Germany). To monitor gene silencing, twelve wells of a 96-wells plate were pooled by lysis with RNABee (Bio Connect, Huissen, the Netherlands) 48 h after transfection and RNA was isolated according the protocol of the supplier. (q)RT-PCR was used to monitor gene silencing as described above. Mean inhibition of mRNA expression was 65% for *BCAR4*, 89% for *ERBB2*, 91% for *ERBB3*, and 62% for *ERBB4* in IPH-926 cells. After five days in culture, a WST-1 proliferation assay (Roche Diagnostics) was performed according to the manufacturer’s recommendations. Lapatinib, a dual-specific ERBB2/EGFR inhibitor [19], was a kind gift from GlaxoSmithKline, Stevenhage, UK. Inhibition experiments were performed as described previously [26].

## Results

### *BCAR4* mRNA expression in human normal tissues and BC cell lines

**Fig 1:**
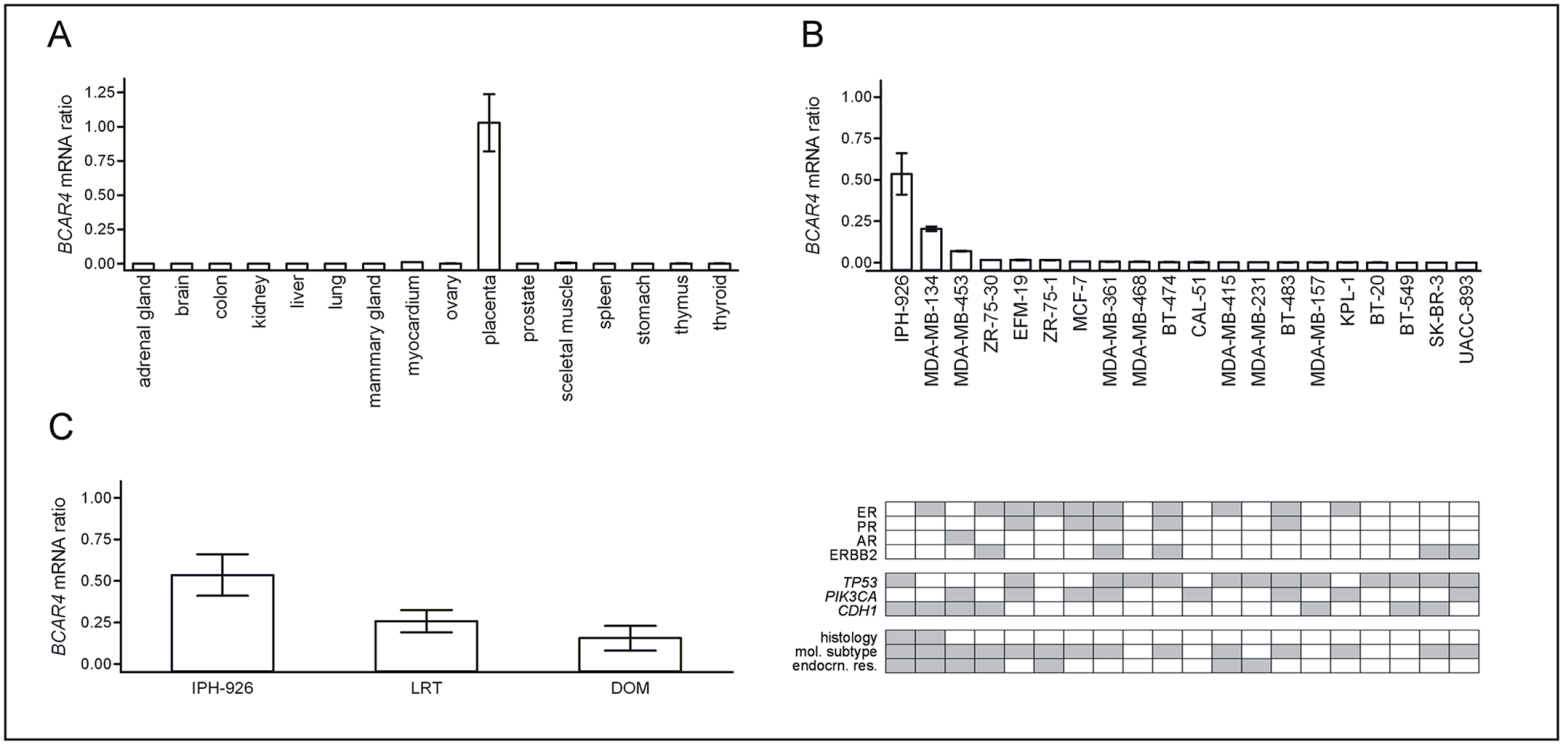
*BCAR4* mRNA expression, as determined by (q)RT-PCR. (A) Analysis of n = 16 human normal tissues. Each tissue was represented by 2–5 specimens from different individuals and expression levels were averaged across these samples. Error bars represent the SEM. (B) Analysis of human BC cell lines. Error bars represent the SEM. The lower panel summarizes cell line characteristics, as retrieved from the literature. Grey indicates: Positive for ER, PR and ERBB2; or mutated/aberrantly methylated for the genes *TP53*, *PIK3CA* and *CDH1*/E-cadherin; or lobular for original tumor histology; or luminal for molecular subtype; or derived after relapse following to clinical anti-hormone therapy (considered as endocrn. res.; endocrine resistance). (C) Analysis of IPH-926 and the corresponding original archival tumor specimens. LRT; locally recurrent tumor, DOM; distant organ metastasis.

Meta-analysis of published microarray datasets has indicated a tissue-specific expression of *BCAR4* in the placenta [10]. We subjected various human normal tissues to *BCAR4* mRNA assessment by quantitative real-time RT-PCR (q)RT-PCR. *BCAR4* mRNA levels were low or undetectable in normal tissues, except for placenta (Fig 1A). In addition, we analyzed a panel of human BC cell lines and thereby identified relatively high *BCAR4* expression in IPH-926 BC cells (Fig 1B). Moderate *BCAR4* expression was detected in MDA-MB-134 and MDA-MB-453. Consistent with previous findings [10], other BC cell lines showed barely detectable or no *BCAR4*. Features of BC cell lines with high/moderate *BCAR4* mRNA included (i) a luminal subtype, (ii) mutation of *CDH1*/E-cadherin and (iii) an origin from endocrine-resistant tumors (Fig 1B, lower panel). For instance, IPH-926 was derived from an endocrine-resistant lobular BC, which had relapsed and converted to an ER-negative phenotype following 5-years-long tamoxifen monotherapy [18, 17, 16, 15]. To rule out that *BCAR4* expression in BC cell lines is an artifact due to *in vitro* cultivation, we analyzed the original clinical tumor specimens corresponding to IPH-926 cells. FFPE tissues of the locally recurrent tumor (LRT) and a distant organ metastasis (DOM) were still available from institutional tissue archives and were subjected to (q)RT-PCR. Compared with IPH-926, the original tumor specimens showed only slightly lower *BCAR4* mRNA levels (Fig 1C). Hence, *BCAR4* mRNA in the tumor has been maintained in the derived BC cell line.

### *BCAR4* mRNA expression is associated high proliferation in primary BCs

**Fig 2:**
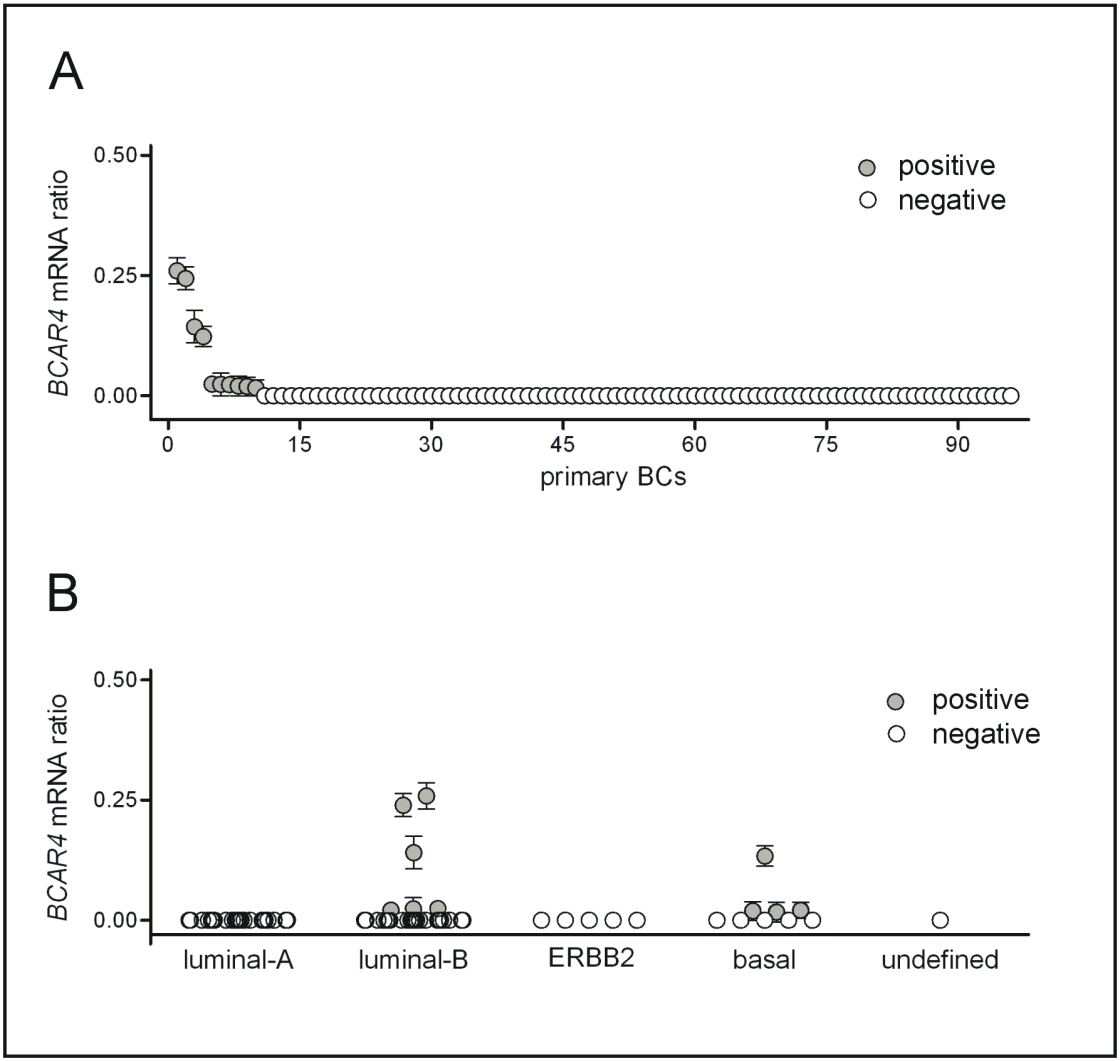
*BCAR4* mRNA expression, as determined by (q)RT-PCR in primary, pre-treatment BCs. Cases are ordered by *BCAR4* mRNA expression level (A), or by molecular subtype (B). Error bars represent SEM.

Next, *BCAR4* mRNA expression was analyzed in a series of primary, pre-treatment BCs. Since BC cell lines with high/moderate *BCAR4* mRNA expression featured *CDH1*/E-cadherin mutations, this tumor series was enriched for lobular carcinomas, which lack E-cadherin due to mutation or epigenetic inactivation (Table 1) [27]. (q)RT-PCR revealed 10.4% (10/96) *BCAR4-*positive cases (Fig 2A). Only 2.1% (2/96) BCs showed *BCAR4* mRNA levels comparable with IPH-926 or MDA-MB-134. *BCAR4-*positive cases were associated with ductal rather than lobular histology, high grade, an ER-negative status and expression of cytokeratin 5/14 (Table 2). Consistently, *BCAR4*-positive cases were also associated with high proliferation, as determined *via* the Ki67 labeling index (Table 2). Classification of tumors by molecular subtype, as defined by a 6-marker panel [23, 24], revealed high *BCAR4* mRNA expression in a small subset of luminal-B BCs, which are associated with endocrine resistance (Fig 2B).

**Table 2:** Relationship between clinicopathological parameters and *BCAR4* mRNA expression in primary BCs.

	*BCAR4*				
	negative		positive		*P* value
**all samples**	86	(90)	10	(10)	
**age**					1.000^a^
<60	22	(92)	2	(8)	
≥60	64	(89)	8	(11)	
**histological type**					**0.021** ^a^
ductal	44	(83)	9	(17)	
lobular	42	(98)	1	(2)	
**histological grade**					**0.007** ^a^
G1/2	64	(96)	3	(4)	
G3	22	(76)	7	(24)	
**pT stage**					0.835^b^
T1	44	(90)	5	(10)	
T2	28	(88)	4	(12)	
T3/4	14	(93)	1	(7)	
**pN stage**					0.706^a^
pN0	47	(92)	4	(8)	
pN1+	29	(88)	4	(12)	
**estrogen receptor**					**0.036** ^a^
negative	10	(71)	4	(29)	
positive	76	(93)	6	(7)	
**progesterone receptor**					0.521^a^
negative	34	(87)	5	(13)	
positive	52	(91)	5	(8)	
**ERBB2**					1.000^c^
0, 1+	78	(89)	10	(11)	
2+/FISH-negative	3	(100)	0	(0)	
3+	5	(100)	0	(0)	
**EGFR**					1.000^a^
negative	84	(89)	10	(11)	
positive	2	(100)	0	(0)	
**CK5/14**					**0.001** ^a^
negative	80	(93)	6	(7)	
positive	6	(60)	4	(40)	
**Ki67 LI**					**0.001** ^b^
0–14	47	(100)	0	(0)	
15–34	27	(82)	6	(18)	
35–100	12	(75)	4	(25)	

^a^Fisher’s exact test,

^b^Chi Square test for trends,

^c^ Fisher’s exact test (0, 1+, 2+/Fish-negative *versus* 3+).

### BCAR4 protein functions as a pro-proliferative factor in BC cells

**Fig 3:**
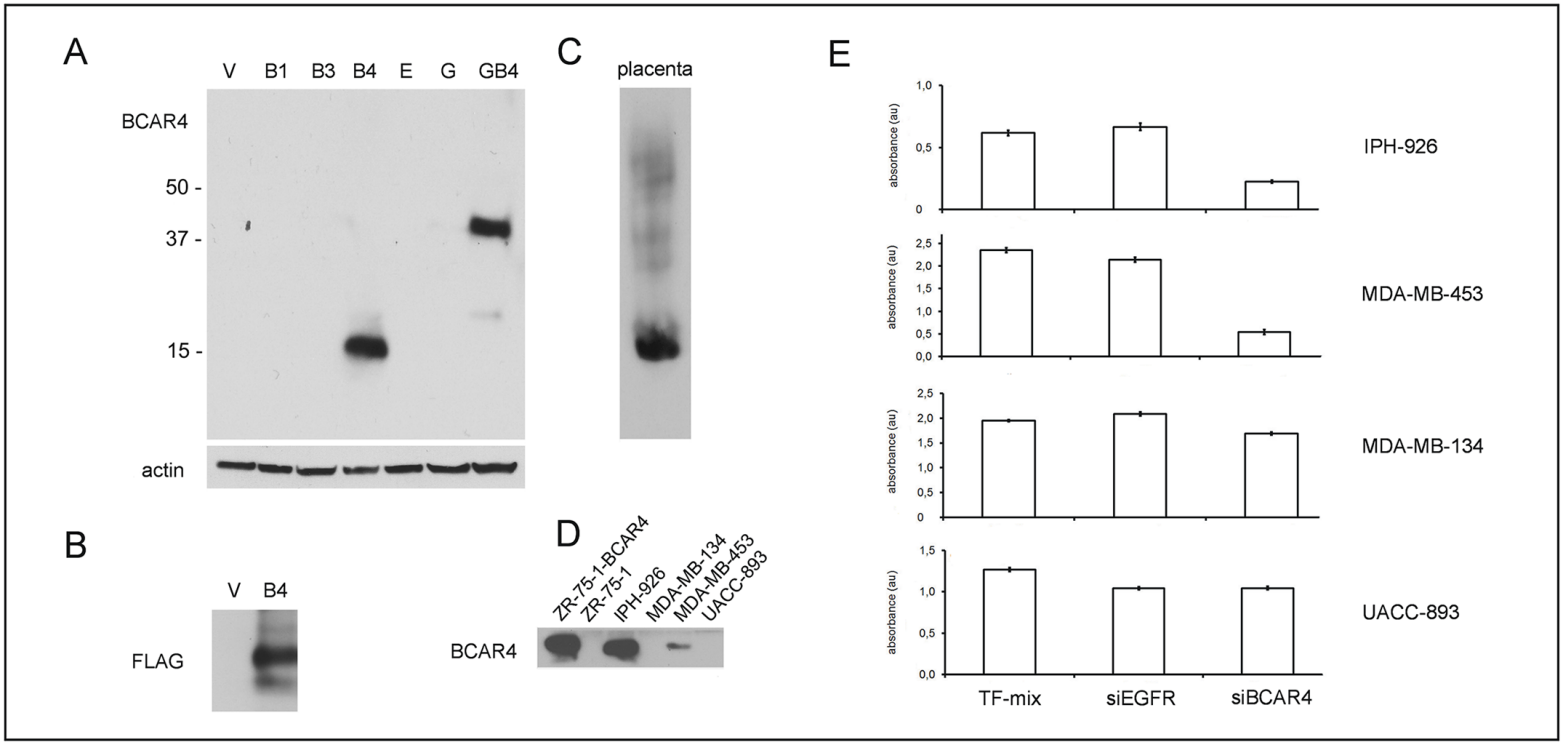
Detection of BCAR4 protein expression with a polyclonal anti-BCAR4 antibody (C78-I97). (A) BCAR4 protein expression in ZR-75-1 BC cells retrovirally transduced with various expression constructs as indicated. V: ZR-75-1 cells transduced with empty vector, B1: BCAR1, B3: BCAR3, B4: BCAR4 E: EGFR, G: EGFP, GB4: EGFP-BCAR4. (B) Western blot analysis of Flag-tagged BCAR4 immunoprecipitated with C78-97 and detected with anti-Flag antibodies. (C) BCAR4 protein expression in human placenta tissue. (D) Western blot analysis of BCAR4 protein expression by immunoprecipitation in human BC cell lines. (E) Human BC cell lines were exposed to siRNAs directed against *BCAR4* (siBCAR4, average of three individual siRNAs), *EGFR* (siEGFR) or to the transfection mix (TF-mix) reagents only. Subsequently, cell proliferation was measured with the WST-1 proliferation assay. Averages of minimal 6 replicates. Error bars represent the SEM.

Several lines of evidence suggest that *BCAR4* encodes a protein [8, 12], but classification as a LncRNA has also been considered [13, 28]. To study BCAR4 protein, we raised a polyclonal antibody, termed C78-I97, against two synthetic human BCAR4 peptides. The specificity of this new antibody was verified by western blot analysis of ZR-75–1 cells transduced with *BCAR4*, *BCAR4-EGFP*, *EGFP*, or with the anti-estrogen resistance related genes *BCAR1*, *BCAR3* and *EGFR* [11]. Only ZR-75-1-*BCAR4* cells showed a band migrating at approximately 13 kd, the predicted size of the putative BCAR4 protein (Fig 3A). ZR-75-1-*BCAR4*-*EGFP* displayed a band migrating at approximately 37 kd. The increased size is due to the EGFP-tag in the BCAR4-EGFP fusion protein (Fig 3A). To further characterize this new antibody, Flag-tagged BCAR4 was immunoprecipitated with C78-I97. Western blot analysis with anti-Flag antibodies detected a protein band of approximately 15 kD, the predicted size of Flag-tagged BCAR4, which further verified the specificity of the C78-I97 antibody (Fig 3B). BCAR4 protein expression was readily detectable by immunoprecipitation with C78-I97 and detection on western blot in human placenta, IPH-926 and MDA-MB-453 cells, but not in MDA-MB-134 and UACC-893 (Fig 3C and 3D). Using ZR-75-1-*BCAR4* cells as a functional model, we have previously shown that *BCAR4* enhances cell proliferation and renders genetically engineered BC cells hormone-independent [10]. Here, we employed IPH-926 as a new model, which has two advantages. First, IPH-926 shows endogenous BCAR4 expression, which provides a physiological cellular background for deciphering BCAR4 function. Second, during *in vivo* tumor evolution, the IPH-926 lineage has lost ER expression, illustrating that hormonal growth regulation was replaced by other pro-proliferative mechanisms [18]. Accordingly, we used siRNA-mediated gene silencing to assess *BCAR4* functions as a pro-proliferative factor in IPH-926 (Fig 3E). Sufficient knockdown of *BCAR4* was verified by (q)RT-PCR (details in materials and methods). As expected, *BCAR4* siRNAs, but not *EGFR* siRNAs, significantly suppressed proliferation of IPH-926 (Fig 3E). A similar effect was observed in MDA-MB-453 cells. In UACC-893 and MDA-MB-134, which lack *BCAR4* mRNA or protein, proliferation was not affected by *BCAR4* inhibition (Fig 3E). Hence, BCAR4 is expressed as a protein and drives proliferation in IPH-926 BC cells.

### Lapatinib counteracts *BCAR4*-driven cell growth

**Fig 4:**
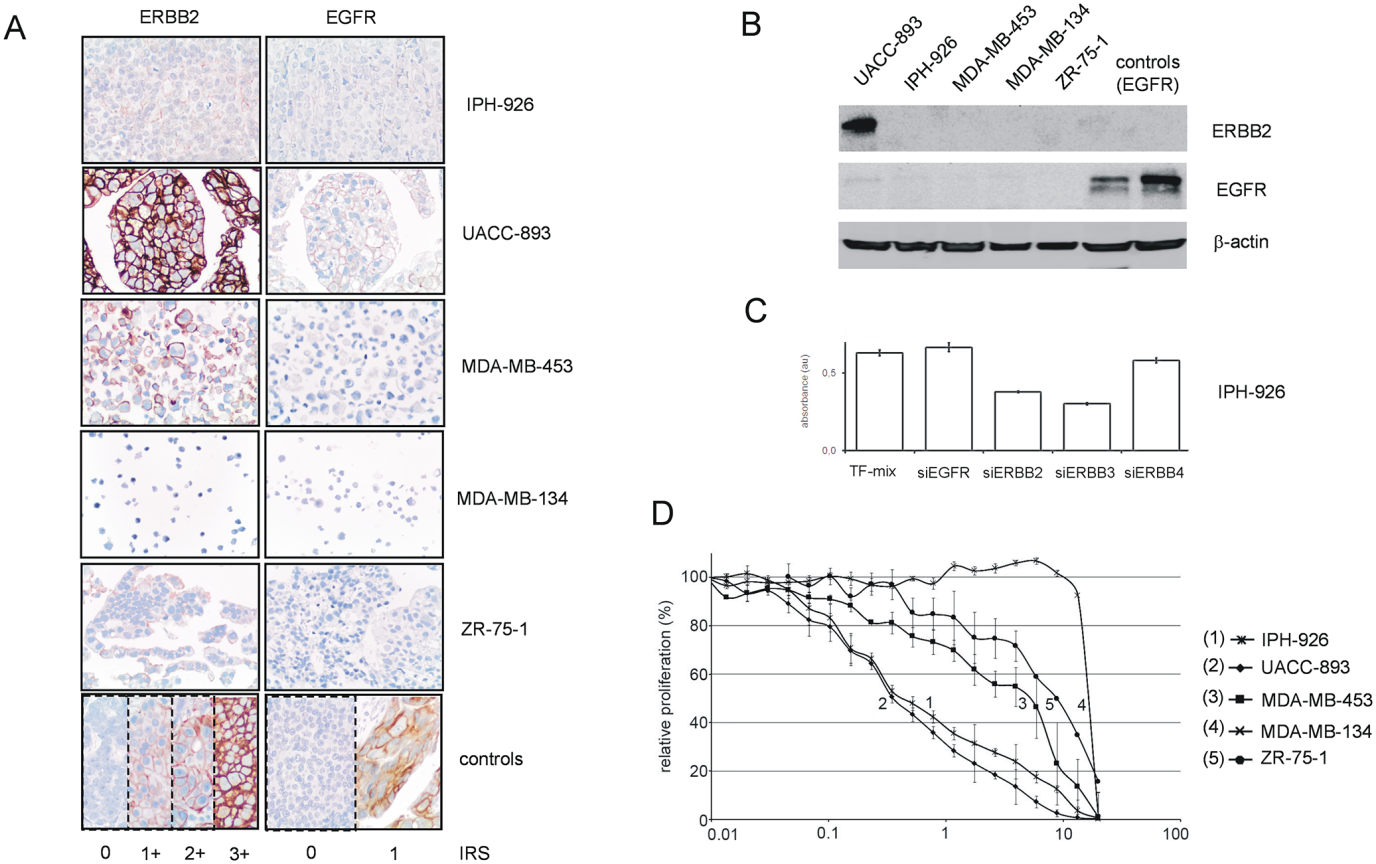
Lapatinib counteracts *BCAR4*-induced cell proliferation. (A) Expression of ERBB2 and EGFR in human BC cell lines, as detected by immunohistochemistry using the clinically validated antibodies 4B5 (ERBB2) and 2.1E1 (EGFR). (B) Expression of ERBB2 and EGFR in human BC cell lines, as detected by western blot using the same immunological reagents. BT20 and MDA-MB-468 BC cells served as positive controls for EGFR [29]. (C) IPH-926 cells were exposed to siRNAs against *EGFR* (siEGFR), *ERBB2* (siERBB2), *ERBB3* (ERBB3), *ERBB4* (ERBB4) or to the transfection mix (TF-mix) reagents only and cell proliferation was measured with the WST-1 assay. Averages of minimal 6 replicates and Error bars representing the SEM are presented. (D) Cells were exposed to various concentrations of lapatinib for five days and cell proliferation was determined with the WST-1 assay. Data are presented as relative proliferation normalized to untreated controls and error bars represent SEM.

Using ZR-75-1-*BCAR4* cells as a model, we have previously shown that *BCAR4* activates ERBB2/3 and its downstream mediators AKT and ERK1/2 by phosphorylation [9, 11]. *BCAR4*-induced cell proliferation was abrogated by knockdown of ERBB2 and ERBB3, suggesting that *BCAR4* acts in an ERBB2/3-dependent manner [9]. High *BCAR4* expression in IPH-926 was an unexpected finding, since these cells are ERBB2-negative as determined with clinically validated detection methods and according to the established scoring system [18, 17, 15, 25]. As shown in Fig 4, IPH-926 cells have very weak ERBB2 protein expression (immunoreactivity score 0 to 1+), which was below detection limit in western blot analysis using UACC-893 cells (amplification of chromosome 17q12) as a positive control (Fig 4A and 4B). Nonetheless, IPH-926 cells were exposed to our previously validated siRNAs against various growth factor receptors, including *ERBB2* and *ERBB3* (Fig 4C). While siRNAs directed against *EGFR* and *ERBB4* had little or no effect, proliferation was suppressed by siRNAs against *ERBB2* and *ERBB3* (Fig 4C). To rule out non-specific effects of siRNAs against *ERBB2/3*, IPH-926 and four other BC cell lines were exposed to lapatinib, a clinically approved small chemical ERBB2/EGFR inhibitor [19]. Consistent with previous studies [29], all cell lines tested for sensitivity to lapatinib were EGFR-negative, except UACC-893, which had minimal EGFR expression (Fig 4A and 4B). Strikingly, IPH-926 (ERBB2 0 to 1+, EGFR 0) was as sensitive to lapatinib as UACC-893 (ERBB2 3+, EGFR 1+) (Fig 4D). The EC_50_ values of lapatinib were approximately 16- to 24-fold lower in IPH-926 and UACC-893 compared with the other BC cell lines. Together, these data imply that BCAR4 acts in an ERBB2/3-dependent manner, even if ERBB2 is expressed at low levels.

## Discussion

The vast majority of breast cancer cases are ER-positive and hormone-dependent. Anti-hormone therapy has thus become the most important, and sometimes the only pharmacological treatment strategy. Due to current clinical trends, an increasing number of BC patients is spared from chemotherapy [30]. However, endocrine resistance is an unresolved problem. The mechanisms underlying endocrine resistance are diverse [4]. This has precluded the establishment of a straight forward set of predictive biomarkers for routine clinical purposes, and the development of targeted therapies to tackle endocrine resistance [6].

We have previously identified the *BCAR4* gene as factor, whose ectopic overexpression can override tamoxifen-induced growth suppression *in vitro* [8]. Follow-up studies revealed that *BCAR4* mRNA levels correlate with tumor aggressiveness in advanced stage BCs and that *BCAR4* acts in an ERBB2/3-dependent manner in ZR-75-1 and MCF7 cells [11,10, 9]. However, human BC cell lines with high endogenous *BCAR4* expression had not been identified [10]. Models for studying *BCAR4* function remained limited to genetically engineered cells, such as ZR-75-1-*BCAR4*, whose physiological relevance was uncertain [10].

The data reported herein are important in several ways. Detailed (q)RT-PCR analyses confirmed that *BCAR4* mRNA expression is restricted to the placenta in human normal tissues. We now successfully identified a human BC cell line with an endogenous, placenta-like high expression of *BCAR4*. This cell line, IPH-926, had been established from an endocrine-resistant lobular BC and can be considered as the physiologic counterpart of the genetically engineered ZR-75-1-*BCAR4* cell model. An anti-BCAR4 antibody was raised and showed for the first time that human *BCAR4* is expressed as a protein. Using IPH-926 cells as a new model and siRNA-mediated silencing of *BCAR4*, we verified that this gene is critical for cell proliferation. Consistent with observations in ZR-75-1, high *BCAR4* expression was associated with sensitivity to ERBB2-inhibition, despite ERBB2 was expressed at very low levels in IPH-926 and ZR-75-1 cells. This substantiates our previous observation, that *BCAR4* acts in an ERBB2-dependent manner [11, 9, 26]. The clinically approved ERBB2/EGFR inhibitor lapatinib emerges as a potential therapeutic alternative for patients with *BCAR4*-positive tumors, showing endocrine resistance. Together, these data firmly establish the role of *BCAR4* in breast cancer biology.

However, this work does not only provide new answers, but also generates new questions. Compared with previous studies in advanced stage BCs, the current work documents a lower rate of primary BC cases with *BCAR4* expression (10% versus 29%) and high expression (2% *versus* 14%). This may be related to the markedly different tumor collections (advanced stage *versus* early stage BCs, enrichment for lobular carcinomas) differences in assays used or the introduction of IPH-926 and human placenta tissue as a benchmark to define high *BCAR4* expression. Future translational studies will have to objectify the real prevalence of high *BCAR4* expression in unselected primary BCs. Even if high *BCAR4* expression should be less common than previously anticipated, *BCAR4* would still represent one of the best-characterized mechanisms of endocrine resistance known so far. Regarding cell signaling, it is interesting that *ERBB2* is expressed at barely detectable or low levels in IPH-926 and ZR-75-1, but still seems critically involved in *BCAR4*-induced cell proliferation. Other studies have also implied, that functionally relevant ERBB2 expression can be below diagnostic thresholds [31, 32]. Clinical ERBB2 testing, which focuses primarily on the identification of an aberrant ERBB2 overexpression, classifies cases without ERBB2 overexpression or amplification as ERBB2-negative. BCs with an aberrant activation of ERBB2, but minimal ERBB2 expression are not identified for targeted therapy [32]. Given that *BCAR4* is associated with ERBB2 activation and sensitivity to ERBB2-inhibition, but is not expressed in the normal mammary gland and most other human normal tissues, it seems that *BCAR4* might be an excellent biomarker to select patients with endocrine resistance for lapatinib therapy. Currently a randomized phase III trial (NSABP B-47) of adjuvant therapy comparing chemotherapy alone to chemotherapy plus trastuzumab in women with node-positive or high-risk node-negative HER2-low invasive BC is ongoing. The aim of that study is to determine whether the addition of trastuzumab to chemotherapy improves disease-free survival in women with resected node-positive or high-risk node-negative breast cancer which is reported as HER2-low by all HER2 testing performed (http://www.nsabp.pitt.edu/B-47.asp)

Our findings that ERBB2 negative, but BCAR4 expressing cells are sensitive to lapatinib-based therapy should be taken into account when interpreting trials exploring the potential of lapatinib to overcome endocrine treatment resistance (https://clinicaltrials.gov/ct2/show/NCT00225758). The rationale to do such trials is primarily based on the inhibitory effects of lapatinib on EGFR and HER2/ERBB2. Our findings suggest that beneficial effects of lapatinib could also be through inhibition of BCAR4 mediated effects, Furthermore, a prospective clinical trial of lapatinib in HER2-negative, BCAR4 positive tumors should be considered.

Recently, the function of *BCAR4* was described as a LncRNA [33]. It was shown that the *BCAR4* RNA is bound to two transcription factors SNIP1 and PNUTS (PPP1R10) activating a non-canonical Hedgehog/GLI2 transcription program. The binding site for SNIP1 is in the 5’ non-coding region of *BCAR4*, the region that is dispensable for induction of tamoxifen resistance [8, 10]. Analysis of GEO expression data of IPH-926, oocyte and placenta with high expression of *BCAR4* showed absence or low expression of *SNIP1*, *PNUTS* and *GLI* target genes, which argues against a major role for the Hedgehog pathway in these tissues. Furthermore introduction of a frameshift by insertion of a thymidine directly following the codon for amino acid four and outside the reported binding domain of PNUTS, abrogated the function of BCAR4 [8]. In addition BCAR4 protein was also demonstrated to be present in the bovine oocyte [12]. Based on these observations, we propose a dual role for BCAR4 as a LncRNA activating the Hedgehog pathway, and as a protein activating the ERBB2 pathway [28, 9]. Further research is needed to resolve these dual functions in the different tissues. Both options may provide attractive possibilities for biological targeted therapy of malignant diseases.
